# Study on the instability mechanism and control technology of narrow coal pillar in double-roadway layout of Changping mine

**DOI:** 10.1038/s41598-024-67412-z

**Published:** 2024-07-19

**Authors:** Tian Cai, Gang Li, Qinghe Yang, Junpeng Zou

**Affiliations:** Institute of Mining Technology, Liaoning Technical University, Fuxin, 123000 China

**Keywords:** Narrow coal pillar, Double-roadway layout, Discrete element, Damage evolution, Engineering, Coal

## Abstract

To address the issue of roadway support failure in narrow coal pillars under dual-lane layout, this study takes the 4309 working face of Changping Coal Mine as the engineering background and employs theoretical calculations, numerical simulations, and on-site monitoring to investigate the instability mechanisms of narrow coal pillars under dual-lane conditions and to optimize technical solutions. The results indicate that the internal stress distribution within the coal pillar is influenced by the advanced support stress, and as the working face advances, the gradually increasing advanced support pressure causes the vertical stress peak within the coal pillar to shift away from the goaf area. Computational analysis reveals that the vertical stress in the top region of a 6 m narrow coal pillar is 38% higher than that in the bottom region, with an average stress of 16 MPa in the coal pillar. The asymmetric high-level stress concentration within the coal pillar significantly affects its stability. A UDEC (Universal Distinct Element Code) model was established to compare four simulation schemes with cut-off angles of 0°, 5°, 10°, and 15°. Based on the analysis of damage parameters and fracture distribution in the narrow coal pillar roadway, it was concluded that the stability is best when the cut-off angle is 10°. The dense drilling cut-off unloading technology was applied to the 4309 working face of the Changping Mine based on the aforementioned research. On-site monitoring results show that the relative deformation of the roof and bottom plates and the two sides of the test section were controlled within 267 mm and 198 mm, respectively, effectively resolving the deformation and instability issues of the narrow coal pillars.

## Introduction

The double-roadway layout system has been promoted in large-scale mines due to its advantages in ventilation and mining succession^1^. However, it is worth noting that in the double-roadway layout system, the coal pillars between roadways are prone to instability and failure due to the excavation of adjacent working face mining roadways, and the stability of coal pillars and overlying strata directly threatens the production safety of adjacent working faces. This is different from the widely used along-goaf roadway excavation technology, where traditional along-goaf roadway excavation refers to excavating roadways along the edge of the goaf with a reserved distance of coal pillar. After the formation of the coal pillar, it is only affected by the mining stress of the working face mining, and the stress conditions of the surrounding rock in the coal pillar roadway are relatively simple. Comparatively, the coal pillars in the double-roadway layout system are successively affected by the influences of the working faces on both sides. Therefore, studying the instability mechanism and control technology of coal pillars in the double-roadway layout system is challenging.

During the mining process, the O-X type rupture is formed in the roof of the mining field, and the arc-shaped triangular block formed by the overlying strata at the edge of the mining field has an impact on the lower structure^2,3^ Many studies have been conducted on the application of along-goaf roadway excavation, along-goaf retained roadway, and other coal pillar-free layout systems by experts and scholars. Xu^4^ provided a detailed explanation of the relationship between the lateral surrounding rock structure of the mining field and the support and offered suggestions for the layout position of narrow coal pillar roadways. Based on this, Wang^5^ classified the lateral surrounding rock structure of the mining field according to the fracture location and concluded that the instability of the coal pillar above the along-goaf roadway is prone to occur when the main roof fracture line is based on the limit equilibrium theory. Subsequently, Qian^6^ conducted calculations and field tests to verify the mining-induced failure zone and the limit equilibrium zone in the coal pillar, based on the theory of ultimate equilibrium. On the other hand, Xia^7^ gathered engineering validation and provided a summary of the roof’s collapse mechanism, stress distribution characteristics, and plastic zone distribution characteristics over the course of the mining operation. Yu^8^ conducted a comprehensive analysis of the stress evolution in the coal pillar’s vicinity and the convergence zone of the tunnel during excavation, leading to roof instability near the tunnel face. They proposed positioning and control strategies for tunnels of this nature. Wang^9^ specifically examined the characteristics of deep-level goaf-side entry failure and highlighted the significant role of increased stress levels in the deep strata as a contributing factor to coal pillar stability. Gao^10^ investigated the fracture process involved in lateral roof failure and elucidated the distinctive features of tunnel failure. Li^11^ provided empirical evidence demonstrating the influence of various fracture locations in the roof, the failure characteristics of filling materials, and the deformation features of goaf-side entries on the location of roof failure in such entries.

Working faces are adopting the dual-heading layout system more frequently as a result of the development of large-scale intelligent mines. Extensive research has been conducted on goaf retention, goaf extraction, and surrounding rock control. However, there is limited research on the stability control of coal pillars and their support structures in the dual-heading layout system under conditions of tunneling, working face mining, and repeated mining disturbances^12,13^. Wu^14^ proposed the reinforcement effect of high-strength support and grouting on coal pillars in gob-side entry retaining. Yao^15^ discussed the influence of advanced support zone anchors on the stress resistance of mining supports. Li^16^ suggested a support form for areas affected by working face mining and tunneling that makes use of I-beams and individual hydraulic supports. These studies considered the impact of dynamic pressure disturbances on support structures but did not extensively investigate the dynamic effects on coal pillars and support structures, referred to as “small structures”, under the disturbance conditions of the dual-heading layout system. The development of coal pillar stability in the dual-heading arrangement system during the course of the disturbance process, however, has not been the subject of nearly any comprehensive study. This is precisely the motivation behind the present study.

Previous scholars have extensively investigated the roof structure, lateral support pressure at the working face, and the disturbance of stress caused by repeated mining. Building upon previous research, this study combines field test results to analyze the influence of dynamic loads from roof fractures on the internal stress distribution of coal pillars. Subsequently, the mechanism of instability in narrow coal pillars at the 4309 working face of the Changping mine is analyzed. After confirming the roof structure as the direct cause of coal pillar instability and failure, a numerical model is established for the research subject. By comparing the deformation and damage of coal pillars under different roof cutting schemes and conducting a comprehensive analysis, reasonable technical parameters are determined. These parameters are successfully implemented in on-site industrial experiments.

## Engineering overview

2.1 Summary of the working face situation.

**Figure 1:**
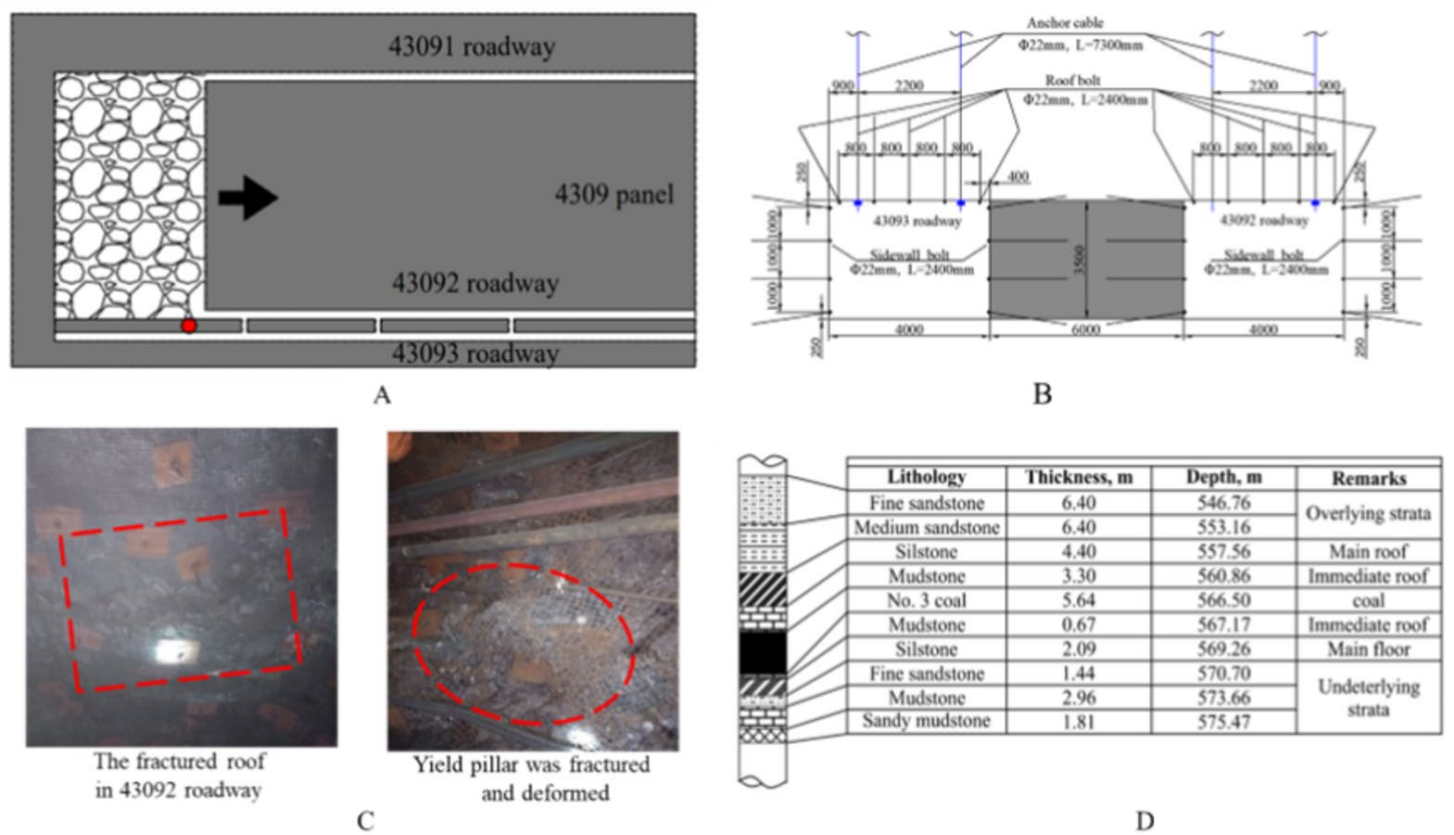
Overview map of the working face.

The Changping Mine 4309 working face is located in the northeastern part of the 4th panel, with a working face length of 200 m and an advance length of 600 m, using the caving method for coal mining. The average thickness of the 3# coal seam where the 4309 working face is located is 5.75 m, which belongs to medium-thick coal seam, with an average overlying strata height of 600 m. The strength of the coal seam and its immediate roof and floor are relatively low. The immediate roof thickness ranges from 1.03 to 10.80 m, with a soft structure, easy water absorption and softening, and low strength. The old roof is sandstone, generally ranging from 0.60 to 13.00 m in thickness, with higher strength, belonging to hard rock. The coal pillar and surrounding rock structure is shown in Fig. 1. The 4309 working face studied in this paper uses a double-roadway layout along the coal seam floor excavation, with a roadway height of 3.8 m, width of 5.8 m. The section coal pillar is located between the 43,092 roadway and the 43,093 roadway, with a width of 6 m, and there is no goaf or special structure nearby. During the mining process, deformation instability of the coal pillar was found, manifested as partial failure of anchor cable support, coal pillar delamination, and coal rib breaking into a connected net pocket shape.

### Support parameters

The roof anchor rods are made of 22 mm diameter, 2400 mm length left-handed non-longitudinal ribbed steel bars. Each row of anchor rods is spaced at 1200 mm intervals, with anchor rod spacing of 1000 mm, and six anchor rods per row. Four anchor rods in the middle of the roof are set vertically to the roof, while those near the roadway shoulder are inclined at 15° towards the rib side. The anchor rod and its anchor tray size are 150 mm × 150 mm × 10 mm, belonging to high-strength arch-shaped trays. They require high-strength self-aligning ball pads, wear-resistant pad rings, and strong anchor rod nuts M24. The anchoring method for the anchor rod is resin-extended anchoring, with an anchoring length of 1200 mm. The roof anchor cables use high-strength, low-relaxation prestressed steel strands with a diameter of 22 mm and a length of 7300 mm, with the tail end using matching high-strength locks. The anchor cable row spacing is 1200 mm, with a spacing of 2400 mm, an anchoring length of 1970 mm, and is set vertically to the roof at a distance of 1700 mm from the edge of both roadway ribs.

The rib anchor rod specifications are the same as the roof anchor rod, with four anchor rods per row, a row spacing of 1200 mm, and a spacing of 950 mm. The top anchor rod is 300 mm from the roof, and the bottom anchor rod is 650 mm from the floor. The anchor rods set in the rib near the top (bottom) plate part are inclined 15° along the vertical rib normal direction. A diamond-shaped mesh made of 10# iron wire is used for rib protection, with a mesh size of 1.35 × 3.7 m, an aperture of 50 mm × 50 mm, one mesh per row, and the mesh is connected by overlapping.

The roadway section is large, with a maximum section of 25–30m^2^. The deformation of the surrounding rock of the roadway is large, especially under the influence of mining. The two ribs of the roadway are approaching 80–100 cm, and the floor heave is 50–80 cm.

## Stress distribution characteristics of coal pillars in different sections

### Lateral support pressure test

In the double-roadway layout method, the coal pillar experiences an evolution from an elastic state to a residual state, which is completely different from the residual state of the coal pillar in the excavation along the goaf. After the excavation of the double-roadway, the surface of the coal pillar between the roadways is damaged under the load of the excavation-induced support, but the central position of the coal pillar is still in the elastic deformation stage, with a certain bearing capacity. As the working face approaches, the support load borne by the coal pillar exceeds its bearing capacity, and the coal pillar as a whole enters the plastic deformation stage, with the vertical stress reaching a high level an d then decreasing. When the coal pillar changes from an elastic state to a plastic state, the roadway experiences large deformation and damage. Therefore, monitoring the stress inside the coal body at different mining stages can more intuitively explain the evolution process of the stress field.

**Figure 2:**
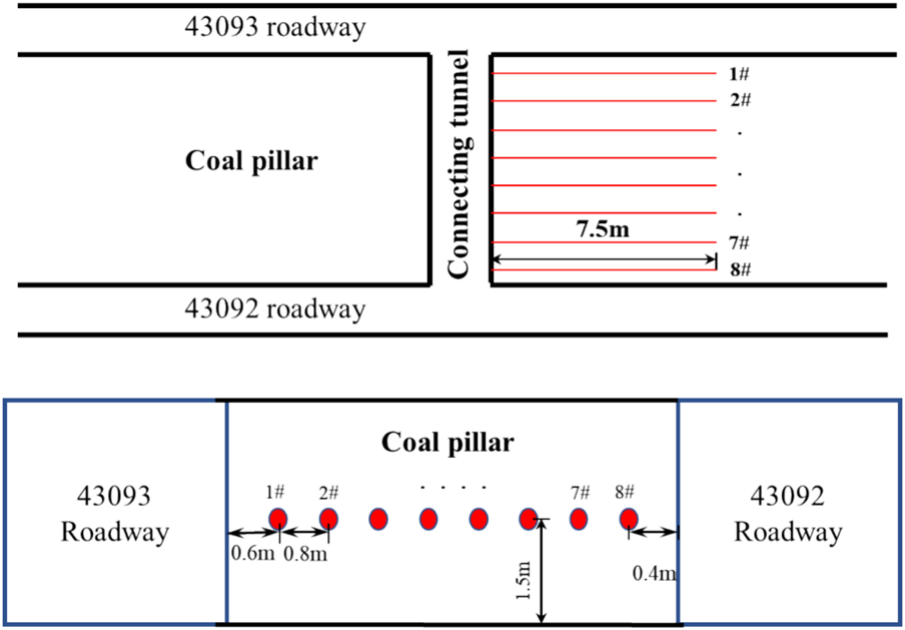
Schematic diagram of station layout.

In this paper, a KSE-III steel string pressure recorder and its data acquisition instrument are used to observe the changes in vertical stress distribution inside the coal pillar caused by the 4309 working face during mining. The test area for lateral support pressure is located in the connecting roadway between 43,092 and 43,093 roadways, and the data acquisition equipment layout is shown in Fig. 2. The stress meter borehole penetrates more than 7.5 m into the coal wall, with a total of 8 stress meters installed. Here, 8 monitoring lines are named in the order of stress meter numbers, and the stress meter spacing from 43,093 to 43,092 roadways is: 0.6 m, 1.4 m, 2 m, 2.8 m, 3.5 m, 4.2 m, 4.9 m, 5.6 m. According to the data statistics of step distance under the 4309 working face in mining engineering, the immediate roof collapse step distance ranges from 18 to 22 m, averaging 22 m; the distance of overlying strata pressure from the old roof above the working face ranges from 32 to 48 m, averaging 40 m; the periodic overlying strata pressure step distance ranges from 18 to 25 m, averaging 22 m, with the peak pressure point of advanced support averaging 20 m from the coal wall. According to the data statistics of surrounding rock deformation, the deformation of the coal pillar increases significantly at a position of 10–15 m ahead of the working face. Based on the above data, screen the results of borehole stress testing to more conveniently observe the internal stress distribution of coal pillars under different mining stages, four time points were selected for data comparison, namely the unmined period of the 4309 working face, the working face advancing to 15 m before the measuring area, the working face advancing to 7 m before the measuring area, and 50 m after the working face mining. The data results intend to illustrate the stress distribution states in three stages: not affected by advanced support pressure, affected by advanced support pressure to a certain extent, and significantly affected by advanced support pressure. For simplicity, they are described as Stage I (not affected by advanced support pressure), Stage II (affected by advanced support pressure to a certain extent), Stage III (significantly affected by advanced support pressure), and Stage IV (residual state stabilized stage).

In Stage I, the working face remains in a pre-mining state. At this point, the interior of the coal pillar is in equilibrium after roadway excavation, resulting in a relatively gentle curve of vertical stress. The internal distribution tends to be symmetric at this stage. The peak vertical stress is recorded at 10.7 MPa, with an average vertical stress of 8.40 MPa across all measurement points. In Stage II, the coal pillar is now 15 m ahead of the advancing working face. Due to the initial influence of advanced support pressure, data from various measurement points indicate an overall increase in stress levels within the coal pillar. The peak vertical stress occurs at Measurement Point 4, reaching 14.1 MPa, with an average vertical stress across all points of 10.16 MPa. In Stage III, with the working face advanced approximately 7 m further, the coal pillar experiences a significant concentration of stress due to increased advanced pressure. The peak vertical stress is observed at Measurement Point 3, reaching 16.9 MPa, with an average vertical stress of 11.81 MPa. Comparatively, in Stage III, the peak stress of the coal pillar increased by 19.8% compared to Stage II, while the average vertical stress increased by 16.2%.In Stage IV, after the working face has pushed 50 m, the coal pillar has reached a new stable state. Data collection equipment at Measurement Points 6–8 is damaged, and no readings are available. The peak vertical stress is observed at Measurement Point 1, reaching 19.2 MPa.

**Figure 3:**
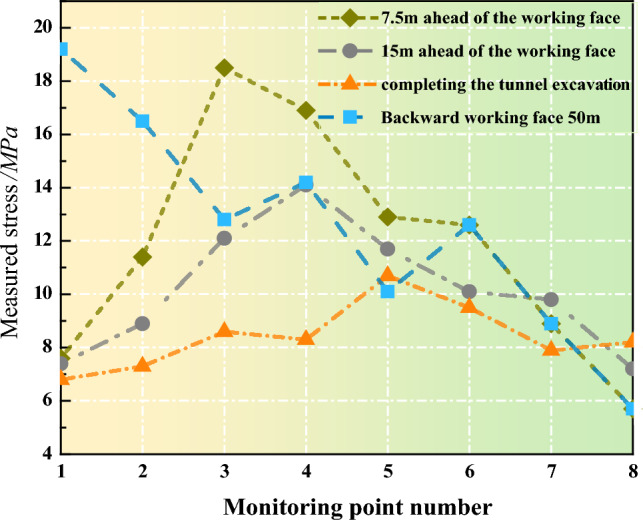
Test results of borehole stress gauges at different periods.

The internal vertical stress distribution of the coal pillar develops from an initially symmetrical form to a single-sided stress concentration form, with the vertical stress peak successively appearing at the 5#, 4#, 3#, and 1# measuring points. As shown in the Fig. 3, the stress change range of the 5# to 8# measuring points is relatively small, while the stress change range of the 1# to 4# measuring points is larger. In the Fig. 3, the values of the 5#, 6#, 7#, and 8# measuring points show a small increase or even a downward trend, which is due to the anchorage failure of the coal pillar rib during the 4309 working face advance. In Stage IV, the data acquisition equipment of the 6#-8# measuring points is damaged, and the readings do not change, but the general stress drop at other measuring points indicates that the coal pillar has entered the residual state. The stress at the 1# measuring point increases by 12.4 MPa, and the roof fracture above it causes the stress to be highly concentrated.

Based on the field test results, the following conclusions can be drawn: the internal stress distribution within the coal pillar is significantly influenced by the advanced support pressure. Under the influence of advanced support pressure, the coal pillar rapidly approaches its load-bearing capacity limit and then rapidly enters a residual state, exhibiting an initial increase followed by a decrease. As the advanced support pressure increases, there is a trend of the peak vertical stress within the coal pillar moving away from the direction of the working face.

### Stress distribution characteristics of coal pillars under mining stress influence

Related research shows that the strength of brittle rocks such as coal under dynamic loading, when subjected only to axial pressure, is lower than the ultimate strength under uniaxial compression. In practical engineering, the coal pillar is approximately subjected to uniaxial compression. In addition to the stress concentration caused by advanced support pressure, the dynamic load released by roof fractures in the mining area also has a significant impact on the stability of the coal pillar.

The study of the seismic energy generated by the key layer rupture of the working face obtains that the disturbance dynamic load genered by the immediate roof rupture during the working face weighting stage^17^ is:

1 $$ \begin{array}{*{20}c} {F_{{\text{d}}} = \rho C_{S} v_{ps} } \\ \end{array} $$

where Cs is the transverse wave propagation speed, and v_ps_ is the maximum vibration speed of the medium particles caused by the transverse wave propagation.

**Figure 4:**
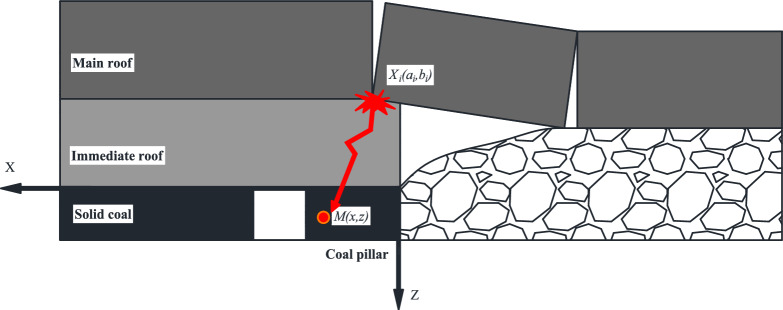
Dynamic load disturbance model of main roof fracture.

As shown in Fig. 4, to study the stability of coal pillars under the influence of dynamic loads, an XZ coordinate system is established, with the edge of the goaf coal seam roof as the origin, and the main roof is assumed to rupture at X_i_ (a_i_, b_i_) to form a dynamic load source F_d_.

The additional stress at any point M (x, z) in the coal pillar under the action of the dynamic load is:

2 $$ \begin{array}{*{20}c} {\left\{ {\begin{array}{*{20}l} {\Delta \sigma_{dxi} = F_{d} \sin^{2} \alpha_{i} } \hfill \\ {\Delta \sigma_{dzi} = F_{d} \cos^{2} \alpha_{i} } \hfill \\ {\Delta {\uptau }_{dxz} = F_{d} \cos \alpha_{i} \sin \alpha_{i} } \hfill \\ \end{array} } \right.} \\ \end{array} $$

Through geometric relationships, it can be known that:

3 $$ \begin{array}{*{20}c} {\left\{ {\begin{array}{*{20}l} {\sin \alpha_{i} = \frac{{\sqrt {\left( {x - a_{i} } \right)^{2} } }}{{\sqrt {\left( {x - a_{i} } \right)^{2} + \left( {z - b_{i} } \right)^{2} } }}} \hfill \\ {\cos \alpha_{i} = \frac{{z - b_{i} }}{{\sqrt {\left( {x - a_{i} } \right)^{2} + \left( {z - b_{i} } \right)^{2} } }}} \hfill \\ \end{array} } \right.} \\ \end{array} $$

Under the disturbance of dynamic loads, the failure of the coal pillar is a process driven by both the static load and the dynamic load increment of mining^18^. Therefore, combining eqs. (2) and (6), the stress increment of the coal pillar after the dynamic load disturbance is:

4 $$ \begin{array}{*{20}c} {\left\{ {\begin{array}{*{20}l} {\Delta \sigma_{x} = \Delta \sigma_{sx} + F_{d} \frac{{\left( {x - a_{i} } \right)^{2} }}{{\left( {x - a_{i} } \right)^{2} + \left( {z - b_{i} } \right)^{2} }}} \hfill \\ {\Delta \sigma_{z} = \Delta \sigma_{sz} + F_{d} \frac{{\left( {z - b_{i} } \right)^{2} }}{{\left( {x - a_{i} } \right)^{2} + \left( {z - b_{i} } \right)^{2} }}} \hfill \\ {\Delta T_{xz} = \Delta T_{sxz} + F_{d} \frac{{\left( {z - b_{i} } \right)\sqrt {\left( {x - a_{i} } \right)^{2} } }}{{\left( {x - a_{i} } \right)^{2} + \left( {z - b_{i} } \right)^{2} }}} \hfill \\ \end{array} } \right.} \\ \end{array} $$

The stress state of any point in the coal pillar is:

5 $$ \begin{array}{*{20}c} {\left\{ {\begin{array}{*{20}l} {\sigma_{x} = \Delta \sigma_{x} + \gamma \lambda \left( {H + z} \right)} \hfill \\ {\sigma_{z} = \Delta \sigma_{z} + \lambda \left( {H + z} \right)} \hfill \\ {T_{xz} = \Delta T_{xz} } \hfill \\ \end{array} } \right.} \\ \end{array} $$

The calculation formulas for the maximum principal stress and minimum principal stress of the coal pillar are:

6 $$ \begin{array}{*{20}c} {\left\{ {\begin{array}{*{20}l} {\sigma_{1} = \frac{{\sigma_{x} + \sigma_{z} }}{2} + \sqrt {\left( {\frac{{\sigma_{x} - \sigma_{z} }}{2}} \right)^{2} + \tau_{xz}^{2} } } \hfill \\ {\sigma_{3} = \frac{{\sigma_{x} + \sigma_{z} }}{2} - \sqrt {\left( {\frac{{\sigma_{x} - \sigma_{z} }}{2}} \right)^{2} + \tau_{xz}^{2} } } \hfill \\ \end{array} } \right.} \\ \end{array} $$

**Figure 5:**
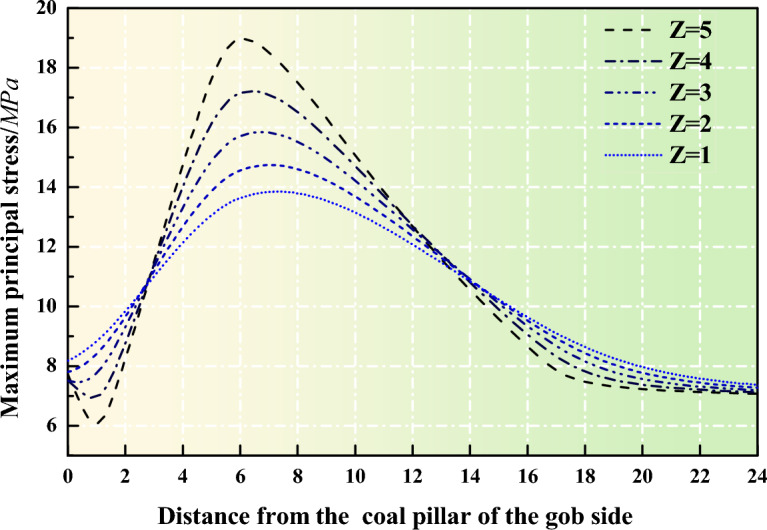
Stress distribution curve inside the coal pillar under dynamic load disturbance.

By substituting the data into the above the equation, the distribution characteristics of vertical stress along the Z-axis of the coal pillar under dynamic loading can be obtained, as shown in Fig. 5. Under the influence of mining-induced stress, the internal stress distribution within the coal pillar changes with its distance from the goaf. It is noteworthy that the curves intersect at distances of 3 m and 14 m from the goaf, indicating that the coal pillar is prone to a low-stress stability state when the distance is less than 3 m. The width of the coal pillar left in the 4309 working face of Changping Mine is 6 m, thus comparing the data with the data at 6 m.At a distance of 6 m from the goaf, the vertical stress at the top of the coal pillar is 38% higher than that at the bottom, with an average stress of 16 MPa. There is varying degrees of stress concentration from top to bottom within the coal pillar, with the highest concentration near the roof being 1.65 and near the floor being 1.23.When the width of the coal pillar is approximately 6 m, the asymmetric high-level stress concentration within the coal pillar greatly affects its stability.

Combining the above analysis, one part of the reason for the failure of roadway support caused by the early establishment of 6 m narrow coal pillars in the 4309 working face can be attributed to the instability caused by local stress concentration within the coal pillar under mining-induced effects. Moreover, the calculation results indicate that the support is more likely to fail due to stress concentration above the coal pillar, which is consistent with the observed asymmetric sinking phenomenon of the gallery roof.

In summary, the failure of narrow coal pillars in the double-roadway layout system can be divided into the following two stages: When the working face advances to the back of the coal pillar, the coal pillar and its roof are locally subjected to stress concentration and damage under the influence of advanced stress, and the internal stress of the coal pillar shows uneven distribution. At this time, the damage of the coal pillar is aggravated. When the working face advances to the coal pillar, the roof fracture releases energy, further aggravating the damage of the coal pillar, and a progressive failure occurs from the top to the bottom of the coal pillar.

## Optimization scheme for roof cutting based on numerical simulation

As can be seen from the previous section, the direct cause of the failure of narrow coal pillars in the double-roadway layout system is the local stress concentration caused by the roof structure. When the bearing capacity of the coal pillar reaches its limit, it enters a residual state, and the energy released during the roof fracture exacerbates the instability and failure of the coal pillar. Based on the above analysis, considering the progress of the working face and the bearing capacity of the coal pillar, the corresponding surrounding rock control technology should start from the perspective of improving the roof structure. The roof-cutting pressure relief technology, as a widely used technology in recent years, can achieve the purpose of improving the stress state of surrounding rock in coal pillar roadways. In the previous production process of Changping Mine, the process of using dense drilling for roof-cutting and pressure relief has been successfully applied and matured. In the 4309 working face, there is sufficient field experience for implementing roof-cutting pressure relief. In this section, numerical simulation is used to demonstrate the roof-cutting scheme of the 4309 working face, and the optimal scheme is obtained through the comparison of the stability control effects of narrow coal pillars.

### Model construction

**Figure 6:**
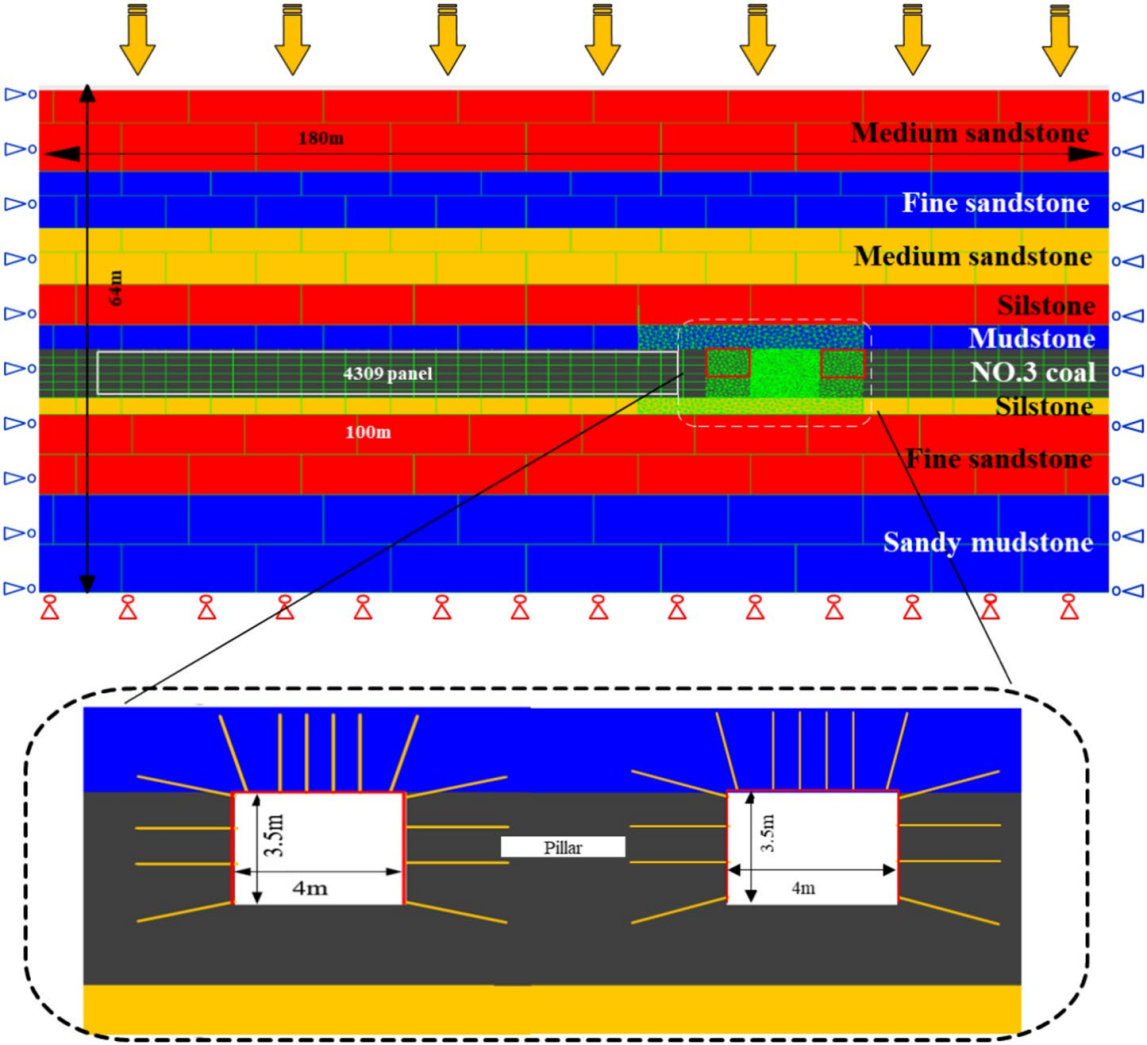
Schematic diagram of the 4309 working face model.

Based on the 4309 working face, a UDEC model is established with a length of 180 m and a height of 64 m. The surrounding structures of the coal pillar are divided into trigon grids to simulate the crack propagation and damage evolution process during the formation of the yielding coal pillar. The model fixes horizontal displacement and vertical displacement at the bottom. As shown in the Fig. 6, the average edge length of these triangular blocks in the coal pillar area is 0.2 m, the roadway section has been outlined with a red rectangle, with the average edge length of the triangular blocks surrounding the roadway coal body being 0.4 m and the average edge length of the triangular blocks in the roof and floor is 0.5 m. To improve modeling efficiency, the surrounding areas of other regions are assigned coarser rectangular blocks with gradually graded increasing edge lengths: 1 m, 2 m, and 4 m, respectively. These block sizes have been proven to be sufficiently fine to represent the mechanical behavior of the coal pillar (Gao et al., 2015). The working face length is set to 100 m in the model, and the bottom and side boundaries are fixed, with a vertical stress of 12.7 MPa applied on the upper boundary of the model.

The size of the small coal pillar in the model between the mining roadway of this section and the mining roadway of the lower section is set to 6 m. The dimensions of the mining roadway are 3500 mm high and 4000 mm wide. The support used in the roadway is anchor cable combined support. There are 4 anchor bolts in each row of roof support, with a spacing of 800 mm between rows. There are 4 anchor bolts in each row of coal rib and wall, with a spacing of 1000 mm * 1000 mm between rib anchor bolts. The initial support resistance is set to 200kN. The numerical model is shown in the following Fig. 6, and the numerical simulation parameters of each rock layer are shown in Table 1.

**Table 1:** Intact rock, rock mass, and mechanical properties.

Rock types and coal	Intact rock		RQD	Rock mass					
	E_r_(GPa)	σ_r_(MPa)		E_m_(GPa)	σ_m_(MPa)				
Medium sandstone	7.33	41.51	86	3.67	27.94				
Mudstone	5.87	33.06	89	3.51	23.48				
Fine sandstone	3.03	15.97	93	3.28	14.47				
Siltstone	3.76	20.98	91	2.05	10.79				
Sandy mudstone	4.64	28.40	95	1.73	9.51				
Coal	2.72	10.93	82	1.12	6.25				

Rock types and coal	Matrix properties		Contact properties						
	Density (kg/m^3^)	E(GPa)	k_n_(GPa/m)	k_s_(GPa/m)	Cohesion(MPa)	Friction angle(°)	Tensile strength (MPa)		
Medium sandstone	2630	9.85	547.9	145.6	7.3	57	3.13		
Mudstone	2610	3.49	501.4	100.3	1.6	39.17	2.94		
Fine sandstone	2770	11.92	397.2	79.5	5.3	35	5.77		
Siltstone	2550	8.81	266.4	63.3	2.8	39.6	4.19		
Sandy mudstone	2300	9.34	337.5	72.5	1.8	44.46	1.78		
Coal	1380	1.02	243.7	45.3	3.8	32	0.28		

Rock types and coal	E(GPa)		Error (%)	UCS(MPa)		Error (%)	BTS(MPa)		Error (%)
	Target	Calibrated		Target	Calibrated		Target	Calibrated	
Medium sandstone	9.85	9.48	−3.75	27.19	26.18	−6.46	4.45	3.48	−21.62
Mudstone	3.49	3.62	3.72	13.13	14.14	−7.69	1.99	2.75	39.10
Siltstone	11.92	12.97	8.80	29.33	31.78	8.35	4.03	3.95	−1.93
Sandy mudstone	8.81	8.04	−8.74	17.34	15.68	−9.57	2.96	1.72	−41.89
Fine sandstone	9.34	9.47	1.39	19.11	18.76	−1.83	2.31	1.77	23.13
Coal	1.02	0.89	−14.55	4.62	4.80	3.89	1.06	0.82	−23.01

### Simulation scheme

According to the rock mass swell theory, considering the roof subsidence and floor heave, the roof cutting height is obtained from the following formula 3:

7 $$ \begin{array}{*{20}c} {H_{{\text{r}}} = \frac{{M - {\Delta }H_{1} - {\Delta }H_{2} }}{K - 1}} \\ \end{array} $$

In the formula: H_r_ is the roof cutting height (m); M is the mining height (m); ΔH_1_ is the roof subsidence (m); ΔH_2_ is the floor heave (m); K is the roof rock mass swell coefficient. Based on the calculation results and construction experience, the roof cutting height is 8 m.

In addition to the roof cutting height, the roof cutting angle also has an important impact on the effect of roof-cutting pressure relief. In this paper, four angles of 0, 5, 10, and 15 are set for comparative analysis of the stability of coal pillars after the working face mining. For convenience of expression, the subsequent text will be described as Scheme 1–4. The numerical simulation in this paper includes the following steps: First, the mechanical parameters obtained from laboratory tests are calibrated numerically, and the geostress is applied and solved in the global model. Second, the grid in the coal pillar roadway area is densified, and after the excavation and support of the roadway, the crack function is used to simulate the roof cutting state. Third, monitoring points are set to record the deformation data of the roadway, and the 4309 working face excavation is carried out. Fourth, adjust the position of the roof cutting angle , and repeat the first three steps to analyze the stability of the coal pillar.

### Parameter calibration

In the discrete element simulation, the mechanical behavior of joints between blocks is manifested by their mechanical parameters, including joint normal stiffness, joint shear stiffness, etc. These parameters still need to be calibrated after laboratory testing to achieve the desired simulation effect.

In this section, the parameters in the model are calibrated^14^, and the rock mechanical parameters, joint micro-parameters, and anchor cable mechanical parameters are corrected separately.

The rock mechanical parameters obtained in the laboratory are corrected to rock mass mechanical parameters to improve the accuracy of model calculation. The RQD method is a common method to estimate the deformation modulus of rock mass. Zhang and Einstein^19^ analyzed the relationship between RQD and Em/Er based on a large number of field monitoring data, and it was verified in the process of adjusting rock mass parameters. In this paper, the RQD value is obtained based on field drilling observation data:

8 $$ \begin{array}{*{20}c} {{\varvec{E}}_{{\mathbf{m}}} /{\mathbf{E}}_{{\mathbf{r}}} = 10^{{0.0186{\varvec{RQD}} - 1.91}} } \\ \end{array} $$

Em and Er in the formula stand for the deformation modulus of unbroken rock and rock mass, respectively. The ratio of Em/Er can be used to modify the uniaxial compressive strength (UCS)^20^:

9 $$ \begin{array}{*{20}c} {\sigma_{m} /\sigma_{r} = \left( {E_{m} /E_{r} } \right)^{n} } \\ \end{array} $$

In the equation, σm and σr are the strengths of rock mass and intact rock, respectively. In addition, the splitting, shearing, sliding, and rotation indexes n are 0.56, 0.56, 0.66, and 0.72, respectively. In this study, the n value is taken as 0.63, considering the complex roof failure process and various failure types^14^. In order to streamline the computation, it is presumed that the transfer function for the ratio of intact rock tensile strength (Tr) to rock mass tensile strength (Tm) is the same^21^. The results are shown in Table 1.

The corrected rock mass property parameters cannot be directly used as the corresponding rock parameters in the model and need to be calibrated numerically to obtain the mechanical parameters of joints and triangular trigon blocks representing the two-dimensional rock mass mechanical properties. Liu^22^ proposed to establish a model for uniaxial compression and Brazilian splitting numerical simulation for numerical calibration.

**Figure 7:**
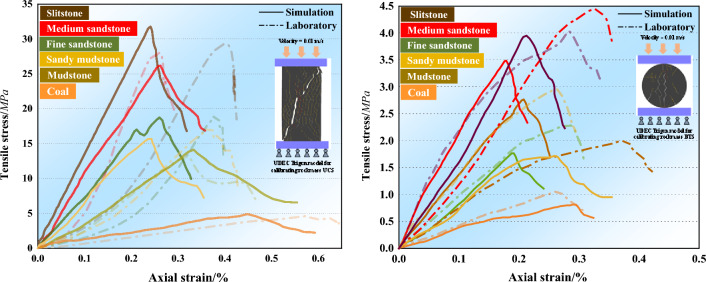
UDEC Trigon models calibration result for UCS(BTS) testing.

A 50 mm wide and 100 mm high unconfined strength test model and a 50 mm diameter Brazilian shear test model are established. The input parameters of the block and contact surface are calibrated by the trial-fitting method; Fig. 7 shows the calibration results for rock and coal, and the micro parameters in Table 1. It can be seen from Table 1 that the maximum error between the unconfined compressive strength and deformation modulus obtained from the numerical simulation model and the indoor test results is less than 15%, and the average error is less than 6.8%, indicating that the calibrated coal and rock mass mechanical parameters in Table 1 are acceptable.

### Results analysis

To quantify the stability of coal pillars in the four simulation schemes, the built-in FISH function of the software is used to record the total length of cracks in the coal pillar after the working face mining, as well as the length of shear and tensile cracks. The damage parameter D is proposed based on Gao^23^:

10 $$ \begin{array}{*{20}c} {D = \frac{{L_{{\text{S}}} + L_{{\text{T}}} }}{{L_{{\text{C}}} }} \times 100\% } \\ \end{array} $$

where L_C_ is the total joint length, L_S_ is the total length of shear cracks, and L_T_ is the total length of tensile cracks.

**Figure 8:**
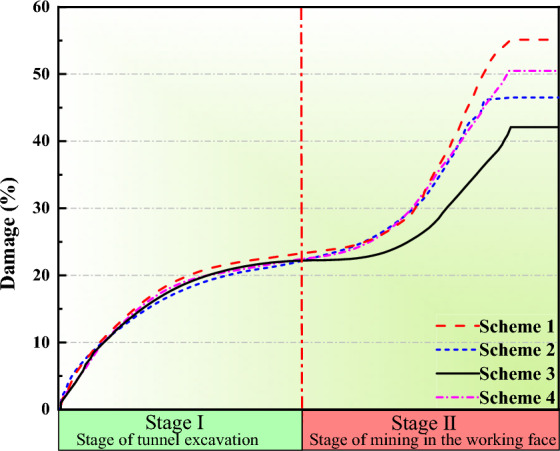
Damage vriables of various numerical simulation schemes.

The numerical model is excavated in two stages: firstly, the roadway is excavated, followed by the excavation of the working face all at once. After completing the calculations, the preprocessed tensile and shear crack data will be exported for further processing. In Fig. 8, each curve corresponds to the variation of damage parameters in numerical models with different roof cutting schemes. As mentioned earlier, damage parameters are used to quantify the abstract concept of coal pillar stability. A higher numerical value indicates a higher degree of damage within the coal pillar, implying poorer stability. During Stage I, the damage parameters (D) in all schemes stabilize at around 20.16%. In Stage II, when sorted in descending order of damage parameters, the schemes are ranked as follows: 55.64% > 53.17% > 46.53% > 42.08%. As coal pillar stability is inversely proportional to damage parameters, among these four simulation schemes, the cutting angle of 10° maintains the best stability of the coal pillar after Stage II.

**Figure 9:**
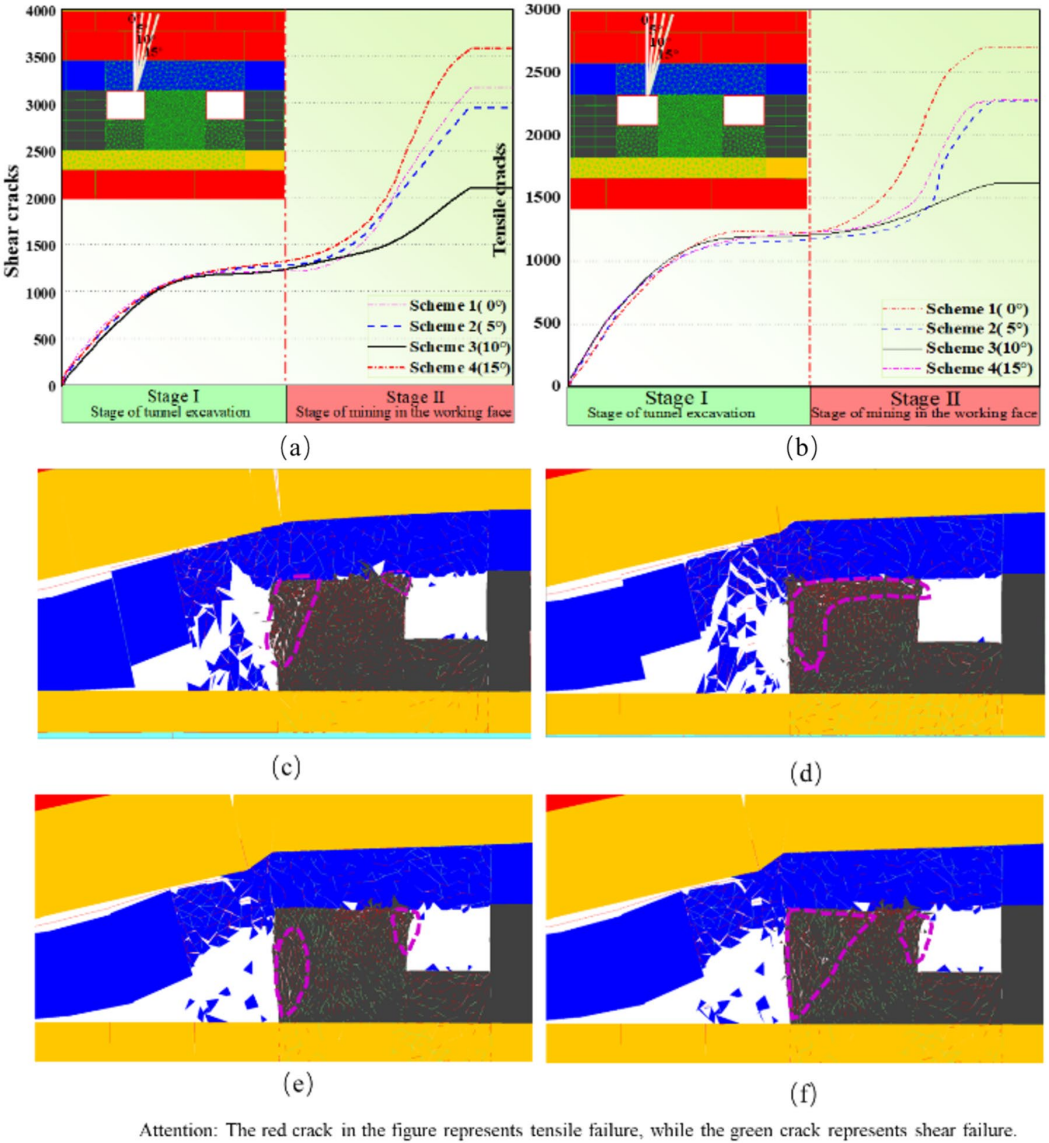
Evolution process of cracks at different roof cutting angles.

As can be seen from the Fig. 9 below, the total length of shear cracks in the coal pillar is significantly higher than that of tensile cracks in Stage I and II, and the overall growth trend is similar, indicating that tensile and shear failure basically develop at the same time. In Stage I, the number of shear and tensile failures is almost the same, and the coal pillar is in a relatively stable stage overall. In Stage II, with the movement of the overlying strata in the mining area, the internal cracks in the coal pillar increase rapidly, and the growth rate of shear cracks is higher than that of tensile cracks. After the movement of the overlying strata in the mining area stabilizes, the cracks stop expanding. The shear cracks in the coal pillar mainly appear in the center of the coal pillar, unlike the tensile cracks, the shear crack extension mode is basically along the vertical direction of the coal pillar. Due to the internal cracks shearing along the rock bridge and penetrating during its development process, the average length of shear cracks is longer, while tensile cracks mainly appear on both sides of the coal pillar, and are more due to the crack expansion after edge unloading, with cross-cracks being the main form. Therefore, it can be judged that shear failure is the dominant failure mode of coal pillar instability.

**Figure 10:**
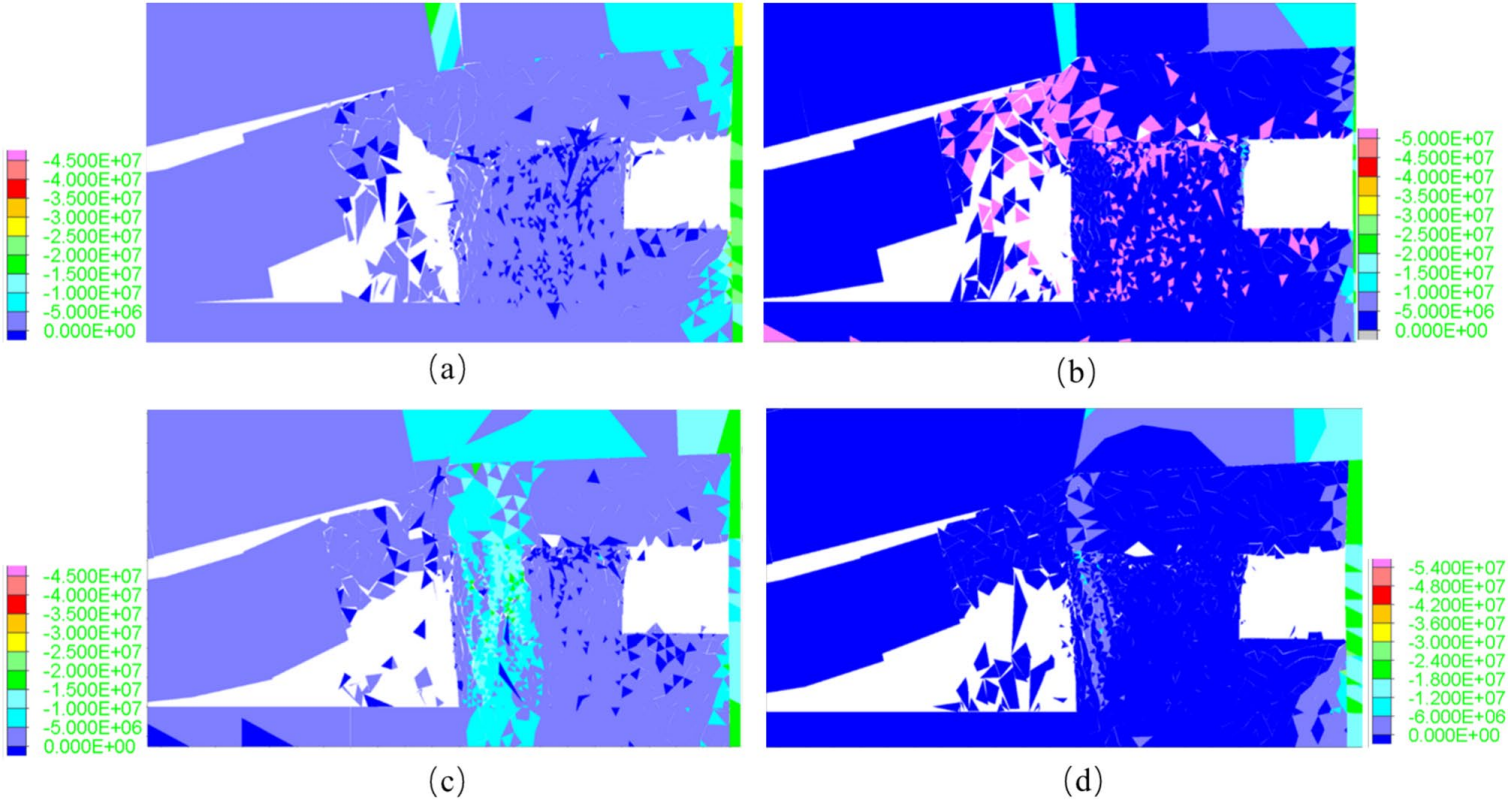
Vertical stress contour of roadway surrounding rock at different roof cutting angles.

As can be seen from the Fig. 10, in Scheme 3, the damage parameter of the coal pillar after the 4309 working face mining is 42.60%, and the growth rate of tensile and shear cracks is significantly lower than that of other schemes. The tensile cracks enter the stable stage earlier than the shear cracks. This means that the crack expansion on both sides enters the stable stage earlier, and at the same time, more shear failures continue to form in the center of the coal pillar. However, according to the comparison of results, there is still a small area of low damage in the center of the coal pillar in Scheme 3. Gao^24^ once proposed that the existence of low damage areas is the key to the stability of the coal pillar.

As shown in Fig. 10, compared to other schemes, the coal pillar roadway exhibits stronger bearing capacity when the cutting angle of the roof is 10°. This indicates that in this scheme, the cutting slots effectively intercept the vertical stress transfer path of the overlying roof, thus the coal pillar does not fail completely, retaining a certain degree of bearing capacity. Considering Fig. 10, the damage to the coal pillar roadway is minor when the roof cutting angle is 10°. After cutting off the vertical stress transfer path of the overlying roof, the shear failure inside the coal pillar is improved. As mentioned earlier, shear failure is the dominant failure mode of coal pillar instability. When the cutting angle of the roof is 10°, the vertical stress state of the coal pillar roadway changes from “compression-shear” to “compression”, reducing the load on the coal pillar and enhancing its integrity.

Simulation results indicate that after the completion of mining at the 4309 working face of the Changping Mine, the primary failure mode inside the coal pillar is “compression-shear”, with a significantly higher number of shear cracks than tensile cracks. Shear failure is the main form of inducing coal pillar failure. When the cutting angle is at a 10° angle with the normal to the roadway roof, after the completion of the working face mining, the coal pillar still retains a partially intact core pressure zone, resulting in better stability control of the coal pillar.

## Stability control technology of narrow coal pillars under mining influence

### Key parameters of narrow coal pillar control technology in double-roadway layout

According to the analysis of the failure characteristics of coal pillars in the previous section, the control technology for narrow coal pillars in the double-roadway layout system should focus on the following two aspects:

1. The edge of the coal pillar is prone to extensive tensile failure due to the influence of advanced stress, so it is necessary to improve the strength of the coal pillar itself and restrain the development of internal cracks.
2. To avoid the fracture of the roof above the narrow coal pillar and strengthen the stress concentration of the coal pillar, it is necessary to improve the roof structure of the mining roadway.

**Figure 11:**
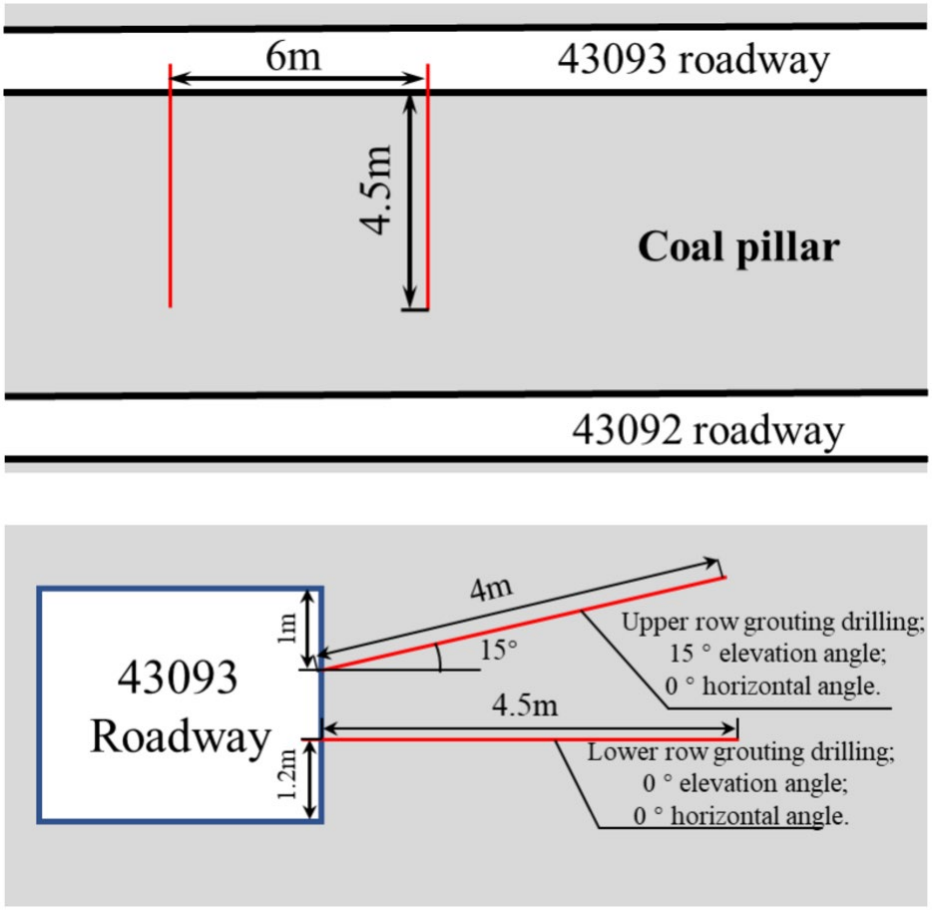
Schematic diagram of drilling arrangement.

Based on the above analysis, the following scheme for controlling the stability of coal pillar roadway in the 4309 working face is proposed:

A. Arrange two rows of grouting holes in the coal pillar, with the hole opening heights shown in the Fig. 11. The lower row of holes is 1.2 m above the roadway floor, and the upper row is 1 m below the roadway roof. The diameter of the grouting holes is 42 mm, and the depth is 4 m. The lower row of holes has a 0° horizontal angle and a 0° pitch angle, with a hole length of 4.5 m and a spacing of 6 m between adjacent holes. The upper row of holes has a construction horizontal angle of 0°, a pitch angle of 15°, a hole length of 4 m, and a spacing of 6 m between adjacent holes.

B. Use the roof-cutting pressure relief technology. Artificial intervention measures to promote roof collapse can effectively release and transfer support stress and improve the stress deformation characteristics of the roadway. On the goaf side of the protected coal pillar, cut the roof holes along the edge of the coal pillar, with a hole depth of 8 m, a spacing of 0.5 m, and a 10° angle between the holes and the roof normal direction. Arrange the roof-cutting holes 60 m ahead of the working face as it advances.

### Field application and deformation statistics

**Figure 12:**
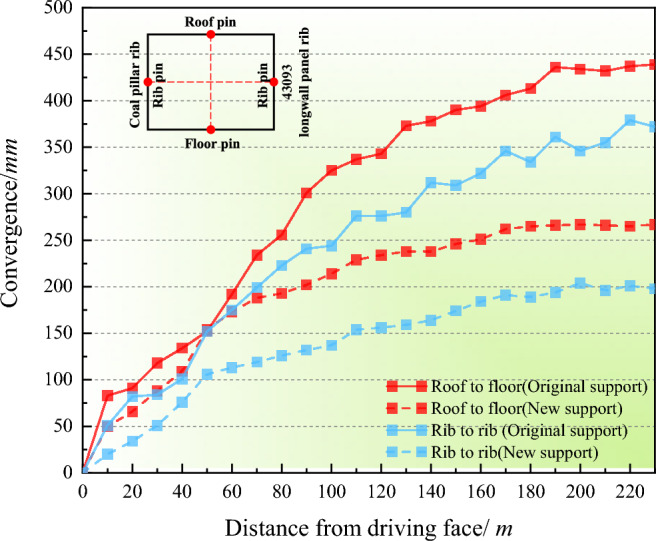
Measured convergences in the 43,092 roadway using roof cutting measures and coal pillar grouting reinforcement.

To verify the applicability of the new surrounding rock stability control method, the cross-layout method was used to monitor the deformation of the surrounding rock during the excavation process on both sides of the 4309 roadway (Fig. 12). Under the support, the surrounding rock of the 43,092 roadway within 32 m in front of the 4309 working face began to deform under the influence of advanced support pressure, while the deformation of the surrounding rock of the 43,092 roadway more than 70 m behind the working face gradually stabilized. The roof-to-floor and rib-to-rib convergences were 267 mm and 198 mm.

The relative convergence of the roof and floor in the original support scheme was 150% higher than that in the new scheme, and the relative convergence of both sides in the original support scheme was 177% higher than that in the new scheme. No rib spalling or roof collapse occurred in the roadway during the use of the new support scheme, and the roof-cutting pressure relief and surrounding rock control effect was significant. The 43,092 roadway, which adopted the optimized support method, met the requirements of transportation and pedestrians and effectively maintained the stability of the 43,092 roadway.

## Conclusion

1. Through stress testing of narrow coal pillars in the 4309 working face of Changping Mine, the stress distribution pattern of coal pillars during mining has been obtained. The internal stress distribution of coal pillars is influenced by advanced support pressure. As the working face advances, the peak vertical stress inside the coal pillar moves towards the non-goaf area. Under the influence of advanced support pressure, the coal pillar rapidly approaches its load-bearing capacity limit and quickly enters a residual state. After the working face advances, roof fractures occur above the coal pillar, exacerbating coal pillar deformation and greatly reducing stability.
2. Stress distribution curves of segmental coal pillars under mining-induced stress were derived. The results show that at a distance of 6 m from the goaf, the vertical stress at the top of the coal pillar is 38% higher than that at the bottom. The average stress within the coal pillar is 16 MPa, and varying degrees of stress concentration are observed from top to bottom. When the coal pillar width is 6 m, the asymmetric high-level stress concentration within the coal pillar significantly affects its stability.
3. A UDEC triangular model was established to simulate different roof cutting schemes. By comparing the damage parameters and crack distributions within the coal pillar, it was concluded that the total length of shear cracks in the coal pillar is higher than that of tensile cracks during both roadway excavation and working face mining stages. Their overall growth trends are similar, indicating that tensile failure and shear failure develop essentially simultaneously, with shear failure playing a dominant role in coal pillar instability. Under a cutting angle of 10°, the coal pillar exhibits the best stability.
4. Combining the mechanism of coal pillar instability and numerical simulation results, support measures were implemented in the 43,092 roadway. Field monitoring shows that the relative deformation of the roadway roof, floor, and sidewalls is reduced by 42% compared to the original support scheme. Moreover, no roof collapse or sidewall spalling occurred during the use of the new support scheme, indicating a significant improvement in surrounding rock control. These support recommendations provide a basis for controlling coal pillar deformation under similar engineering geological conditions.

## Data Availability

The datasets generated during and/or analyzed during the current study are available from the corresponding author on reasonable request.
